# Inhibition of breathing after surfactant depletion is achieved at a higher arterial PCO_2 _during ventilation with liquid than with gas

**DOI:** 10.1186/1465-9921-6-24

**Published:** 2005-03-04

**Authors:** Esther Rieger-Fackeldey, Richard Sindelar, Anders Jonzon, Andreas Schulze, Gunnar Sedin

**Affiliations:** 1 Department of Women's and Children's Health, Section for Pediatrics, Uppsala University, Uppsala, Sweden; 2 Department of Neuroscience, Division of Physiology, Uppsala University, Uppsala, Sweden; 3 Department of Obstetrics and Gynecology, Division of Neonatology, Klinikum Grosshadern, Ludwig Maximilian University, Munich, Germany

## Abstract

**Background:**

Inhibition of phrenic nerve activity (PNA) can be achieved when alveolar ventilation is adequate and when stretching of lung tissue stimulates mechanoreceptors to inhibit inspiratory activity. During mechanical ventilation under different lung conditions, inhibition of PNA can provide a physiological setting at which ventilatory parameters can be compared and related to arterial blood gases and pH.

**Objective:**

To study lung mechanics and gas exchange at inhibition of PNA during controlled gas ventilation (GV) and during partial liquid ventilation (PLV) before and after lung lavage.

**Methods:**

Nine anaesthetised, mechanically ventilated young cats (age 3.8 ± 0.5 months, weight 2.3 ± 0.1 kg) (mean ± SD) were studied with stepwise increases in peak inspiratory pressure (PIP) until total inhibition of PNA was attained before lavage (with GV) and after lavage (GV and PLV). Tidal volume (V_t_), PIP, oesophageal pressure and arterial blood gases were measured at inhibition of PNA. One way repeated measures analysis of variance and Student Newman Keuls-tests were used for statistical analysis.

**Results:**

During GV, inhibition of PNA occurred at lower PIP, transpulmonary pressure (Ptp) and Vt before than after lung lavage. After lavage, inhibition of inspiratory activity was achieved at the same PIP, Ptp and Vt during GV and PLV, but occurred at a higher PaCO_2 _during PLV. After lavage compliance at inhibition was almost the same during GV and PLV and resistance was lower during GV than during PLV.

**Conclusion:**

Inhibition of inspiratory activity occurs at a higher PaCO_2 _during PLV than during GV in cats with surfactant-depleted lungs. This could indicate that PLV induces better recruitment of mechanoreceptors than GV.

## Background

Partial liquid ventilation (PLV) combines liquid ventilation and gas ventilation (GV). Perfluorocarbon is administered to the trachea in a volume equivalent to the pulmonary functional residual capacity, and ventilation is maintained with conventional gas ventilation of the liquid-filled lung [1]. The improvement of gas exchange during PLV is mainly due to recruitment of collapsed alveoli [2], decreased physiological shunting and increased compliance [3].

During breathing of gas the rate and depth of breathing is influenced by mechanoreceptors in the lung [4-6], and by peripheral and central chemoreceptors, which modulate the phrenic motoneurone output representing central inspiratory activity [7]. An increase in tidal volume and flow rate during mechanical ventilation with gas results in a decrease in magnitude or duration of the phrenic nerve signal [8,9], with absence of that response after vagotomy [8]. It has been shown that inhibition of inspiratory activity can be achieved with air with high frequency positive pressure ventilation (HFPPV) [10] at ventilatory frequencies of 60–100/min and with different positive end-expiratory pressures (PEEP) and insufflation periods in animals [11] and in humans [12] at normo-ventilation. To achieve inhibition of phrenic nerve activity (PNA) during ventilation with air at lower ventilatory frequencies than 60, a lower arterial PCO_2 _and a higher pH will be needed [11].

No studies have been presented concerning PNA during PLV, but it has been shown in studies of animals that spontaneous breathing can take place during PLV with beneficial physiological effects [13,14]. Inhibition of PNA can thus provide a physiological setting at which ventilatory pressures, volumes and arterial blood gases can be compared during GV and during PLV in surfactant-depleted animals.

This study was therefore undertaken to determine whether inhibition of PNA can be achieved at the same airway or transpulmonary pressures during GV and PLV and to find out at what levels of arterial blood gases and pH inhibition occurs with these modes of ventilation in cats with healthy and surfactant-depleted lungs.

## Methods

### Animal Preparation

Juvenile cats (n = 9; mean ± SD; age 3.8 ± 0.5 months, weight 2.3 ± 0.1 kg) were anaesthetised with chloroform, placed in a supine position and endotracheally intubated (tube 4.0 mm inner diameter). The tube was then connected to an infant ventilator (Stephanie^®^, F. Stephan Biomedical Inc., Gackenbach, Germany) and the animal was placed on assist control (A/C) ventilation during the surgical procedures with the following settings: peak inspiratory pressure (PIP) 1.0 kPa, positive end-expiratory pressure (PEEP) 0.3 kPa, inspiratory time (Ti) 1 sec, respiratory rate (RR) 30/min.

The right femoral vein and artery were dissected and catheters were inserted with the tip of each catheter placed in the thorax close to the heart. Anaesthesia was continued with 0.72% α-chloralose (Sigma-Aldrich Chemie GmbH, Steinheim, Germany) (50 mg/kg) and maintained at regular intervals via the venous line. A continuous infusion of 10% glucose (2/3) and 5% 0.6 M sodium bicarbonate (1/3) was given at a rate of 6.4 mL/kg/h (7.15 mg/kg/min of glucose) through the venous catheter throughout the experiment. The arterial line was used for continuous monitoring of blood pressure and intermittent determination of blood gases (Acid-Base Laboratory ABL 505^®^, Radiometer Corp., Copenhagen, Denmark). The cat's core body temperature, measured as deep rectal temperature, was maintained at 38°C by a heating blanket and an overhead warmer.

A pretracheal midline incision was performed for preparation of the trachea, the oesophagus and both phrenic nerves. A tight ligature was tied around the trachea in order to prevent air leakage around the tube. An 8 French catheter with an oesophageal balloon (40 × 7.5 mm; flat frequency response up to 5 Hz) was inserted into the distal part of the oesophagus and a ligature was softly tied around the oesophagus to avoid air entrance into the stomach [15]. Both phrenic nerves were exposed and the connective sheath was removed. The intact right phrenic nerve was then placed on two platinum electrodes. For reasons of isolation the phrenic nerves, the electrodes and the dissected area were submerged in mineral oil [16].

### Measurements and data collection

Airflow was measured by a sensor placed between the endotracheal tube connector and the Y connector of the tubing circuit of the Stephanie^® ^infant ventilator. This sensor is a pneumotachometer with the dynamic properties of an original Fleisch 00 pneumotachograph, but with less dead space (0.6 ml) and resistance (1.1 kPa/l/s at 5 L/min) [17]. Airflow was calibrated with a precision flowmeter (Timeter RT 200 ^®^, Timeter Instrument Corporation, Lancaster, PA, USA). Airway pressure (P_aw_) was measured at the connector of the endotracheal tube. Oesophageal pressure (P_oes_) was recorded from the oesophageal balloon catheter by a pressure transducer (Druck Ltd. Transducer, Leicestershire, UK) and, like P_aw_, was calibrated with a water manometer. Arterial blood pressure and heart rate were measured using the same type of transducer (Druck Ltd. Transducer, Leicestershire, UK) connected to the arterial catheter with the tip of the catheter at the same level as the transducer. Continuous recordings of arterial blood pressure and heart rate were made with a polygraph recorder (Recorder 330P, Hellige AG, Freiburg, Germany).

PNA was amplified, filtered and rectified with a Neurolog system^® ^(Digitimer Research Instrumentation Inc., Welwyn Garden City, Hertforshire, UK; preamplifier NL 103, AC-amplifier NL 105, filters NL 115). The rectified nerve signal was fed to a spike trigger to produce spikes of uniform amplitude (Digitimer 130^® ^and Spike Trigger NL 200, Digitimer Research Instrumentation Inc., Welwyn Garden City, Hertforshire, UK) and subsequently integrated by a resistance-capacitance low-pass filter with a leak (time constant 250 ms), providing a moving time average of PNA. Monitoring of the signals was achieved by means of an oscilloscope (Tektronix Inc., Portland, Oregon, USA).

Signals of airflow and P_aw _were obtained directly from the analogue outlets of the ventilator. Together with signals of P_oes _and the PNA they were transferred to an analogue-digital converter and recorded on disk at a sampling rate of 10 kHz per channel by a data acquisition system (Windaq Pro+^®^, Dataq Instruments Inc., Akron, OH, USA). Compliance and resistance values were given by the ventilator.

### Protocol

The cats were kept ventilated with air using A/C ventilation and the ventilation was adjusted so that normal arterial blood gases were achieved. The cats were then treated with endotracheal continuous positive airway pressure with 0.3 kPa PEEP in order to monitor and record the spontaneous breathing activity of each cat. Pressure-controlled mechanical ventilation with sinusoidal inspiratory waveform was then initiated with the following settings: RR 60/min; Ti 0.33 sec; PIP 0.8 to 1.0 kPa using a PEEP of 0.5 kPa. PIP was adjusted so that blood gas values were in a normal range. The fraction of inspired oxygen was kept at 0.21. PIP was then gradually increased until rhythmic PNA disappeared. Three breaths after inhibition of PNA, data from 20 consecutive breaths were recorded and arterial blood gases were analysed.

Thereafter lung lavage was performed by filling the lungs with warmed saline solution (37.5°C, 30 mL/kg) through a funnel connected to the endotracheal tube. Very gentle chest compressions were performed to allow the saline to be well distributed, before it was removed by suctioning. This procedure was repeated 7 to 8 times and mechanical ventilation was provided in between the lavage procedures. After a 30-minute period of stabilisation on mechanical ventilation (PIP/PEEP 3.0/0.5 kPa, RR 60/min, Ti 0.33 sec, FiO_2 _1.0), ventilation was increased until PNA was inhibited. Airway pressures were then recorded and arterial blood gases and pH were measured again.

In the next step a bolus of 30 ml/kg prewarmed (38°C) perfluorocarbon (PFC) (Perfluorodecaline^®^, F2 chemicals Ltd, Preston, Lancashire, UK) was instilled into the endotracheal tube via an adapter with a side port. Instillation of PFC into the lung was performed within 10 minutes during pressure-controlled ventilation (PIP/PEEP 3.2/0.5 kPa, RR 60/min, Ti 0.33, FiO_2 _1.0). Sufficient filling was ascertained by disconnecting the endotracheal tube from the ventilator circuit at the end of the filling procedure and observing to see that a meniscus was present in the endotracheal tube at end-expiration. If no meniscus could be observed prior to recording, additional PFC was instilled. After a stabilisation period of 10 minutes, the cats were studied with the same protocol during PLV as during GV, but with an FiO_2 _of 1.0 and a PIP adjusted to blood gases in the normal range.

Data on PNA could be recorded and the whole protocol could be completed in all nine cats. Lavage and instillation of PFC were well tolerated, with no coughing or gasping. No bradycardia or arterial hypotension occurred during the procedure.

The experiments were performed at the Biomedical Centre of Uppsala University and were approved by the Uppsala University Animal Research Ethics Board (No. C224 / 0).

### Data Analysis and Statistics

Windaq Playback^® ^Software (Dataq Instruments, Inc., Akron, OH, USA) was used to review the recorded signals. Analysis was done by means of Windaq Playback^® ^and Excel^® ^(Office 2000, Microsoft Corporation, USA). For statistical evaluation, Sigmastat^® ^(SPSS Inc, IL, USA) was used.

The amplitude of the integrated PNA was monitored and inhibition of spontaneous breathing activity was defined as total disappearance of PNA, i.e. total inhibition of inspiratory activity.

Gas flow, P_aw _and P_oes _were measured at peak inspiratory pressures. The airflow signal was integrated to obtain tidal volumes (V_t_) at different PIPs. Transpulmonary pressure (P_tp_) was calculated as Paw – Pes. Lung compliance (C_L_) is given as the ratio of V_t _over P_tp_. In three cats an endotracheal tube leak of > 20% of the tidal volume was found, and in those cats no volume values were therefore calculated and consequently no compliance values can be given.

After inhibition of PNA, the 20 breaths were evaluated at the three settings studied, i.e. during GV with a normal lung, and during GV and PLV after surfactant depletion. Data are presented as mean ± SD or median and 25th and 75th percentiles. One way repeated measures analysis of variance (ANOVA) or RM ANOVA on ranks was performed to test for differences between the three groups. Student-Newman-Keuls tests were applied for comparisons between two groups when a difference was detected by ANOVA. The level of significance was set at p < 0.05 in all tests. An a posteriori power analysis revealed that the study had a power of 99% to detect a difference in PIP between healthy and surfactant-depleted lungs during GV, and of 100% to detect such a difference between healthy and liquid-filled lungs (n = 9). The power values for detecting differences in tidal volume between the same groups were 98% and 61% respectively (n = 6).

## Results

**Figure 1:**
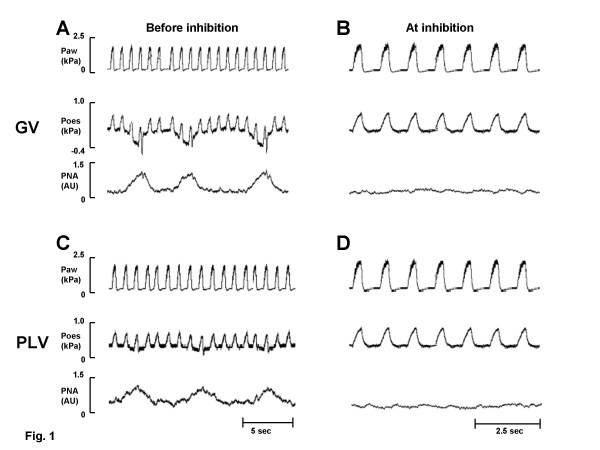
**Recording before and after inhibition of breathing. **Recording of airway pressure (P_aw_), oesophageal pressure (P_oes_) and phrenic nerve activity (PNA) before inhibition of spontaneous breathing in a representative cat after lung lavage during gas ventilation (GV) (A) and during partial liquid ventilation (PLV) (C), and at inhibition during GV after lung lavage (B) and during PLV (D).

Inhibition of PNA could be achieved in all cats during GV and PLV both before and after lavage at the applied ventilatory frequency of 60/min, insufflation time 0.33% of the period time and PEEP of 0.5 kPa. Figure 1 shows examples of recordings before and at inhibition of spontaneous breathing after lavage during GV (A and B) and during PLV (C and D) in one representative cat.

### Ventilatory parameters and lung mechanics

**Figure 2:**
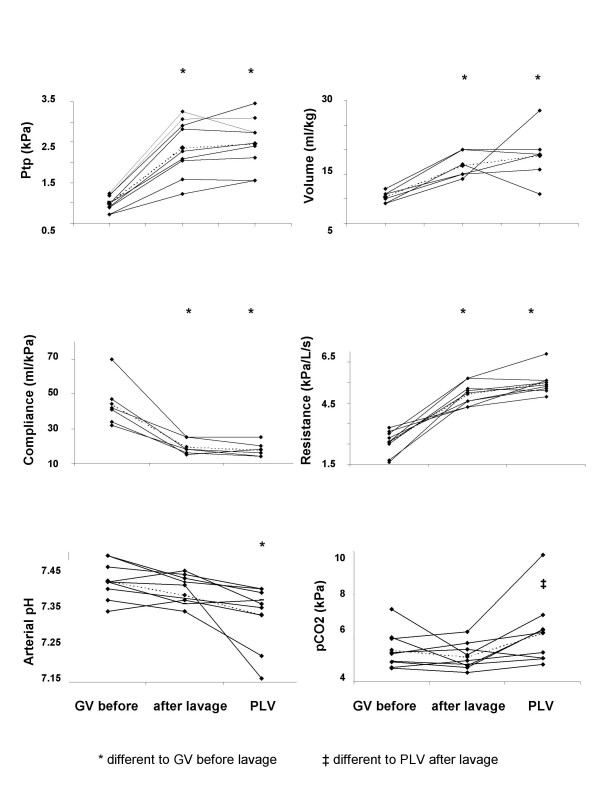
**Lung mechanics and blood gases. **Multipanel figure showing (A) transpulmonary pressure; (B) tidal volume; (C) lung compliance; (D) resistance; (E) arterial pH; (F) arterial pCO_2 _during gas ventilation (GV) before lavage and during GV and partial liquid ventilation after lavage in each cat (unbroken lines) and as mean (broken line).

Inhibition of PNA occurred at a lower PIP (Table 1), a lower P_tp _and lower tidal volumes (Table 1 and Fig. 2) before lavage than after lavage. Compliance at inhibition of inspiratory activity was higher before than after lavage (Table 1 and Fig. 2). Resistance was lower before than after lavage during GV.

**Table 1:** Ventilatory parameters, lung mechanics and arterial blood gases at inhibition of spontaneous breathing

	**GV**		**PLV**	
	**before lavage**	**after lavage**	**after lavage**	**p**

**PIP (kPa)**	1.3 ± 0.2	2.8 ± 0.6*	2.9 ± 0.6*	*<0.001
**P_tp _(kPa)**	0.98 ± 0.2	2.36 ± 0.7 *	2.46 ± 0.6*	*<0.001
**V_t _(ml/kg)**	10 ± 1.2	17 ± 2.6*	19 ± 5.6*	*<0.02
**C_L _(ml/kPa)**	41.5 [34;47]	18 [16;25]*	17 [14;20]*	*<0.05
**Resistance (kPa/L/s)**	2.58 ± 0.59	4.94 ± 0.54*	5.49 ± 0.59 *‡	*<0.001 ‡ = 0.038

**pH**	7.42 ± 0.05	7.38 ± 0.07	7.33 ± 0.8*	* = 0.008
**PaCO_2_, kPa**	5.5 ± 0.9	5.2 ± 0.6	6.3 ± 1.7‡	‡ = 0.027
**PaO_2_, kPa**	14.1 ± 1.8	11.0 ± 6.0	29.2 ± 17.1*‡	* = 0.01 ‡ = 0.01
**BE**	1.71 ± 1.47	-2.08 ± 2.97*	-1.89 ± 3.95*	*<0.001

* different from GV before lavage ‡ different from GV after lavage

Mean ± SD; RM ANOVA and Student-Newman-Keuls Tests or RM ANOVA on ranks with 25 and 75 percentiles (for compliance values only)

PIP = peak inspiratory pressure; P_tp _= transpulmonary pressure; V_t _= tidal volume; C_L _= lung compliance; BE = base excess

After lavage, PIP and P_tp _were similar at inhibition during GV and during PLV. After lavage, compliance at inhibition remained the same during GV and PLV and resistance was lower during GV than during PLV (Table 1 and Fig. 2).

**Figure 3:**
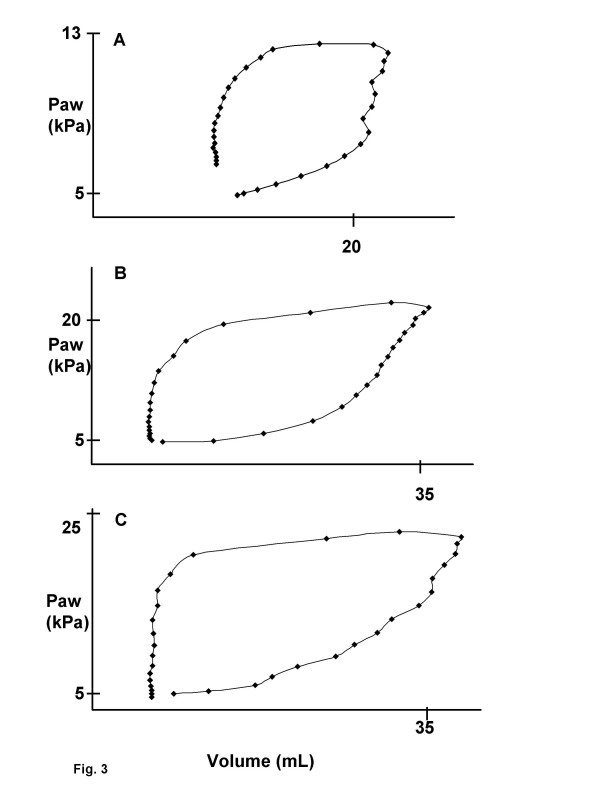
**Pressure-volume curves. **Pressure-volume curve during (A) gas ventilation (GV) before lavage and (B) during GV and partial liquid ventilation (C) after lavage in a representative cat.

Figure 3 shows the pressure-volume loops at inhibition during GV before lavage and during GV and PLV after lavage in a representative cat. The loop obtained before lavage shows the highest compliance, whereas the loop obtained during PLV after lavage shows the highest resistance.

### Arterial blood gases

Before lavage, inhibition of PNA during GV occurred at an arterial pH of 7.42, which did not differ significantly from the post lavage arterial pH at inhibition of PNA. There was no statistically significant difference in arterial pH at PNA inhibition between GV and PLV. At inhibition of PNA the arterial PCO_2 _was lower during GV before lavage than after lavage, but was higher during PLV than during GV after lavage (Table 1 and Fig. 2). Arterial PO_2 _was at a level which provided sufficient oxygenation at all settings (Table 1).

## Discussion

This study shows that in cats ventilated with gas, inspiratory activity is inhibited at higher peak airway pressures and tidal volumes after lung lavage than before. In cats with surfactant-depleted lungs, inhibition of inspiratory activity occurs at about the same airway pressures and tidal volumes during GV and during PLV, but at higher arterial PCO_2 _during PLV than during GV.

PLV with perfluorocarbon is a method of ventilatory support introduced by Fuhrman in 1991, wherein gas is ventilated into a partially liquid (perfluorocarbon) filled lung (1). PLV has been shown to decrease the alveolar surface tension mainly in dependent parts of the lung, resulting in alveolar recruitment and reduced ventilation-perfusion mismatch, thereby improving gas exchange and lung mechanics [18]. These beneficial effects of PLV have been demonstrated not only in animal models of respiratory distress and meconium aspiration syndrome [19,20], but also in adults and newborn infants with severe respiratory distress syndrome [21,22].

In the present study a ventilatory strategy of a moderate PEEP (0.5 kPa) and positive pressure ventilation at 60/min was chosen in a model of surfactant depletion to simulate a relevant clinical situation in which lung recruitment and possibly low tidal ventilation could be promoted. The point of inhibition of PNA represents the time point at which central inspiratory activity ceases and at which all spontaneous breathing activity has disappeared completely. It has been used as a point of comparison between different ventilatory patterns [11].

Lung compliance did not differ between PLV and GV in surfactant-depleted lungs, but resistance was higher during PLV, as reported elsewhere [2]. This might not represent a real increase in resistance of the airways, but is more likely due to higher inertia of the liquid than of the gas.

In this study all experiments were performed in the same order and time sequence, i.e. first GV in the healthy lung, and then GV and PLV in that order in the surfactant-depleted lung. We avoided randomisation of the order of GV and PLV in the surfactant-depleted lung, as that approach would have meant a much longer period of mechanical ventilation in the group randomised to PLV as the first part of the protocol, to allow evaporation of the perfluorocarbon.

In cats with normal lungs the pulmonary stretch receptor (PSR) activity is similar during GV and PLV, indicating that there is no extensive stretching of the lung during PLV [15]. On the other hand, the impulse frequencies of PSRs are higher at the start of inspiration with PLV than with GV at the highest insufflation pressures used [15]. This might also be the case when the lung has been lavaged and surfactant-depleted.

In animals with surfactant-depleted lungs, which may be partially atelectatic, mechanoreceptors in some well-ventilated areas may be active, whereas other receptors in atelectatic areas may not give any impulses. In the present study all receptors which were active during GV were also active during PLV. The study showed that during GV inhibition of PNA occurred at much higher airway pressures after than before lung lavage, but at similar arterial blood gases, findings which might be due to an altered stretch receptor input from, for example, areas that are surfactant-depleted and/or partially atelectatic. As instillation of perfluorocarbon might exert an effect similar to that of surfactant on lavaged lungs, increased mechanoreceptor discharge during PLV due to increased stretch receptor activity might explain why PNA inhibition occurs at a higher arterial PCO_2 _during PLV than during GV. This possibility is supported by the finding that administration of surfactant increases the activity of mechanoreceptors in surfactant-depleted animals [23]. It is unlikely that a high arterial PO_2 _during PLV influences the respiratory drive.

## Conclusion

Higher airway pressures are needed to achieve inhibition of inspiratory activity during GV in animals with surfactant-depleted lungs than in animals with normal lungs. After surfactant depletion, inhibition of inspiratory activity during PLV occurs at about the same peak inspiratory and end-expiratory pressures and tidal volume as during GV. Inhibition of inspiratory activity occurs at a lower arterial pH and a higher arterial PCO_2 _during PLV than during GV in animals with surfactant-depleted lungs, which might be explained by recruitment of pulmonary stretch receptors during PLV. This may be a reason why inhibition of spontaneous breathing is more easily achieved during PLV than during GV in animals with surfactant-depleted lungs.

## List Of Abbreviations

**PNA**, phrenic nerve activity

**GV**, gas ventilation

**PLV**, partial liquid ventilation

**PIP**, peak inspiratory pressure

**V_t_**, tidal volume

**HFPPV**, high frequency pressure ventilation

**PEEP**, positive end-expiratory pressure

**A/C ventilation**, assist/control ventilation

**Ti**, inspiratory time

**RR**, respiratory rate

**P_aw_**, airway pressure

**P_oes_**, oesophageal pressure

**P_tp_**, transpulmonary pressure

**C_L_**, lung compliance

**RM-ANOVA**, one way repeated measures analysis of variance

## Competing Interests

The authors declare that they have no competing interests.

## Authors' Contributions

ERF participated in designing the study, was involved in the preparation and care of the animals, was responsible for the acquisition and analysis of the data and drafted the manuscript. RS participated in the design of the study, was responsible for the preparation of the animals, was involved in the acquisition and analysis of the data, and revised the manuscript. AJ participated in the design of the study, was responsible for the preparation of the animals and for the neurophysiological recordings, and revised the manuscript. AS made substantial contributions to the data collection and their interpretation, and revised the manuscript. GS conceived of the study and its design, performed the lavage and PFC instillation procedures, helped to interpret the data, and revised the manuscript. All authors read and approved the final manuscript.
